# Risk factors for prolonged surgical duration of tracheobronchial foreign body removal in children: a single-center retrospective study

**DOI:** 10.3389/fped.2024.1508702

**Published:** 2025-01-07

**Authors:** Zhe Su, Jianmin Zhang, Zhengzheng Gao, Fang Wang, Lijing Li, Zenghua Xu, Heqi Liu, Fuzhou Zhang

**Affiliations:** Department of Anesthesiology, Beijing Children’s Hospital, Capital Medical University, National Center for Children’s Health, Beijing, China

**Keywords:** children, tracheobronchial foreign body, surgical duration, risk factor, retrospective study

## Abstract

**Object:**

This study aims to analyze the clinical characteristics of children with tracheobronchial foreign body and to investigate the factors influencing the surgical duration of rigid bronchoscopic foreign body removal under general anesthesia.

**Methods:**

We retrospectively identified 421 children diagnosed with tracheobronchial foreign body undergoing rigid bronchoscopy between January 2020 and December 2021. A comprehensive analysis was conducted on patient demographics, including age, weight, gender, American Society of Anesthesiologists (ASA) physical status classification, foreign body type and location, duration of foreign body retention, preoperative symptoms, signs, imaging findings, tracheobronchial manifestations observed during bronchoscopy, and surgical durations. Statistical analysis utilized both univariate and multivariate linear regression models to assess factors influencing the surgical duration of tracheobronchial foreign body removal in children.

**Results:**

The mean age of children with tracheobronchial foreign body was 1.59 years (1.32, 2.04). The male-to-female ratio was 1.8:1, and the ASA physical status classification was predominantly ASA II (96.7%). Organic foreign body accounted for 94.8% of cases, with 91.7% located unilaterally. Univariate and multivariate linear regression analyses revealed that ASA III, pulmonary rales, and the presence of one, two, or three specific tracheobronchial manifestations observed during bronchoscopy—including mucosal hyperemia and edema, purulent exudate, and granulation tissue—were independent risk factors associated with prolonged surgical duration for foreign body removal (*P* < 0.05).

**Conclusion:**

ASA III, pulmonary rales, and various tracheobronchial manifestations observed during bronchoscopy are significant risk factors associated with prolonged surgical duration for foreign body removal in children.

## 1 Introduction

Tracheobronchial foreign body aspiration is a common and critical condition in pediatrics, primarily affecting children aged 1–3 years, with a higher incidence observed in boys (1, 2). The standard clinical approach for managing this condition is the removal of foreign body using rigid bronchoscopy under general anesthesia. Surgical duration is a key variable in predicting the incidence of complications during and after surgery (3). Prolonged surgical duration is often associated with an increased risk of complications, including perioperative hypoxemia, laryngeal edema, and tracheal obstruction (4, 5).While numerous studies have explored the clinical characteristics of tracheobronchial foreign body aspiration, there is a notable lack of research focusing on the factors influencing the surgical duration of rigid bronchoscopic foreign body removal under general anesthesia. To address this knowledge gap, we conducted a retrospective analysis of clinical data from 421 children undergoing rigid tracheobronchial foreign body removal under general anesthesia between January 2020 and December 2021. Our analysis aimed to summarize the clinical characteristics and patterns observed, employing linear regression analyses to identify factors relevant to the surgical duration of rigid bronchoscopic foreign body removal in children.

## 2 Materials and methods

### 2.1 Research subjects

We selected a total of 421 children diagnosed with tracheobronchial foreign body at Beijing Children's Hospital between January 2020 and December 2021, all of whom underwent rigid bronchoscopic foreign body removal. The surgeons and anesthesiologists involved in these cases each had clinical experience ranging from 5 to 10 years. This deliberate selection aimed to minimize heterogeneity among the clinicians concerning qualifications, experience, and skills during surgical procedure and anesthesia management.

### 2.2 Research methods

#### 2.2.1 Data collection

Basic Information: We extracted age, weight, gender, and ASA physical status classification from electronic medical records.

Foreign Body-Related Information: Data collected included the type (organic or inorganic), location (main trachea, left bronchus, right bronchus, bilateral bronchi), and duration of retention (time from foreign body aspiration to removal by bronchoscopy).

Preoperative Information: We documented specific symptoms, signs, and imaging manifestations (e.g., fever, cyanosis, pulmonary rales, tri-retraction sign—an indicator of significant airway obstruction characterized by noticeable retraction in the suprasternal notch, supraclavicular fossae, and intercostal spaces during inspiration—emphysema, pneumonia).

Intraoperative Information: We recorded tracheobronchial manifestations observed during bronchoscopy (mucosal hyperemia and edema, purulent exudate, granulation tissue) and the surgical duration (from insertion of the rigid bronchoscope to removal of all foreign bodies).

#### 2.2.2 Data analysis

We conducted multivariate linear regression analysis to examine the factors affecting the surgical duration of rigid bronchoscopic foreign body removal in children. Additionally, we compared surgical durations among children with one, two, or three tracheobronchial manifestations observed during bronchoscopy (mucosal hyperemia and edema, purulent exudate, granulation tissue) to those with no tracheobronchial manifestations.

### 2.3 Statistical methods

Statistical analysis was performed using SPSS version 25.0. Normality was assessed using histograms and the Kolmogorov-Smirnov test. Measurement data were presented as mean ± standard deviation or median and quartile [M (Q1, Q3)], as appropriate. Enumeration data were expressed as frequency and percentage [*n* (%)], with descriptive analysis conducted for each observation index. Univariate linear regression analysis was applied to identify factors affecting surgical duration. Independent variables with *P* < 0.1 in the univariate analysis were included in the multivariate linear regression model for a comprehensive analysis to identify risk factors for surgical duration. Predictors tested included gender, weight, ASA physical status classification, pulmonary rales, tri-retraction sign, and the presence of one, two, or three tracheobronchial manifestations observed during bronchoscopy (mucosal hyperemia and edema, purulent exudate, granulation tissue). A significance threshold of *P* < 0.05 was established for group comparisons.

## 3 Results

### 3.1 Demographic and clinical characteristics

Table 1 illustrates the demographic characteristics, foreign body attributes, preoperative, and intraoperative conditions of the 421 children included in this study. The median age was 1.59 years (1.32, 2.04), and the median weight was 11.50 kg (10.50, 13.00). Among the subjects, 268 (63.7%) were male, with the majority classified as ASA physical status II (96.7%). Organic foreign body constituted 94.8% of cases, with the left bronchi (46.8%) and right bronchi (44.9%) being the most common locations. The median duration of foreign body retention was 24.00 h (12.00, 72.00). Preoperative special symptoms included fever (1.9%) and cyanosis (2.1%). Specific preoperative signs were noted in a few cases, such as pulmonary rales (3.6%) and the tri-retraction sign (0.7%). Preoperative imaging manifestations included obstructive emphysema (18.1%) and bronchopneumonia (5.2%). Tracheobronchial manifestations observed during bronchoscopy included airway mucosal hyperemia and edema (8.8%), purulent exudate (9.5%), and granulation tissue (7.1%). The median surgical duration for rigid bronchoscopic foreign body removal under general anesthesia was 10.00 min (7.00, 15.00).

**Table 1:** Demographic and clinical characteristics of children with tracheobronchial foreign body (*n* = 421).

Characteristics	*N* (% or IQRs)	Characteristics	*N* (% or IQRs)
Age (year)	1.59 (1.32, 2.04)	Preoperative special symptoms	
Weight (kg)	11.50 (10.50, 13.00)	Fever	8 (1.9)
Gender		Cyanosis	9 (2.1)
Males	268 (63.7)	Preoperative special signs	
Females	153 (36.3)	Pulmonary rales	15 (3.6)
ASA		Tri-retraction sign	3 (0.7)
II	407 (96.7)	Preoperative imaging manifestations	
III	14 (3.3)	Emphysema	76 (18.1)
Type of foreign body		Pneumonia	22 (5.2)
Organic matter	399 (94.8)	Intraoperative tracheobronchial manifestations	
Inorganic matter	22 (5.2)	Mucosal hyperemia and edema	37 (8.8)
Location of foreign body		Purulent exudate	40 (9.5)
Left bronchi	197 (46.8)	Granulation tissue	30 (7.1)
Right bronchi	189 (44.9)	Surgical duration(min)	10.00 (7.00, 15.00)
Duration of foreign body retention (hour)	24.00(12.00, 72.00)		

IQRs, interquartile ranges; ASA, American Society of Anesthesiologists.

### 3.2 Univariate and multivariate analysis of surgical duration for tracheobronchial foreign body removal

**Figure 1:**
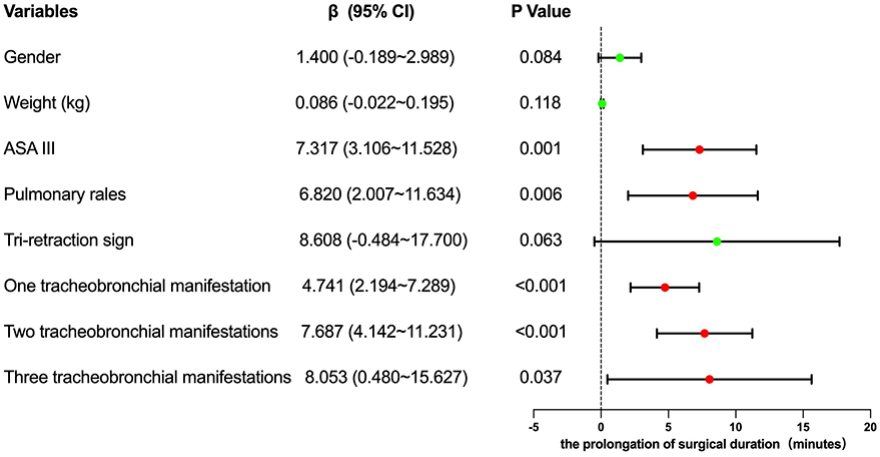
Forest plot for independent risk factors affecting the prolongation of surgical duration of foreign body removal. This forest plot illustrates the impact of various independent risk factors on the prolongation of the surgical duration required for foreign body removal. The risk factors analyzed include gender, weight, ASA III status, pulmonary rales, tri-retraction sign, and the presence of one, two, or three tracheobronchial manifestations. Each risk factor is associated with a beta coefficient (*β*), a 95% confidence interval (CI), and a *p*-value indicating statistical significance. Factors with red dots indicate significantly prolonged surgical duration.

The results of the univariate analysis indicated that the *P*-values for patients' gender, weight, ASA physical status classification, pulmonary rales, tri-retraction sign, mucosal hyperemia and edema, purulent exudate, and granulation tissue were all less than 0.1, warranting their inclusion in the multivariate analysis (Table 2). The multivariate analysis revealed that ASA III, pulmonary rales, and the presence of one, two, or three tracheobronchial manifestations observed during bronchoscopy were independent risk factors associated with prolonged surgical duration (*P* < 0.05). Specifically, children with ASA III had a surgical duration approximately 7.3 min longer than those with ASA II. Additionally, children presenting with preoperative pulmonary rales had a surgical duration about 6.8 min longer than those without these symptoms. Surgical duration increased by 4.7 min with one tracheobronchial manifestation, 7.7 min with two manifestations, and 8.0 min with three manifestations (Figure 1).

**Table 2:** Univariate & multiple linear regression analyses of factors affecting surgical duration of tracheobronchial foreign body removal in children.

Factors	Univariable model			Multivariable model		
	*β*	95% CI	*P*	*β*	95% CI	*P*
Gender	1.676	−0.031∼3.383	0.054	1.400	−0.189∼2.989	0.084
Age (year)	0.320	−0.138∼0.778	0.170	–	–	–
Weight (kg)	0.107	−0.010∼0.225	0.073	0.086	−0.022∼0.195	0.118
ASA III	6.766	2.212∼11.320	0.004	7.317	3.106∼11.528	0.001
Type of foreign body	−0.119	−3.825∼3.587	0.950	–	–	–
Location of foreign body						
Left bronchi	−0.899	−2.550∼0.751	0.285	–	–	–
Right bronchi	0.498	−1.160∼2.155	0.555	–	–	–
Duration of foreign body retention(hour)	0.003	−0.002∼0.008	0.182	–	–	–
Preoperative special symptoms						
Fever	−1.159	−7.199∼4.881	0.706	–	–	–
Cyanosis	−1.758	−7.458∼3.942	0.545	–	–	–
Preoperative special signs						
Pulmonary rales	13.079	8.810∼17.348	<0.001	6.820	2.007∼11.634	0.006
Tri-retraction sign	13.375	3.653∼23.096	0.007	8.608	−0.484∼17.700	0.063
Preoperative imaging manifestations						
Emphysema	−0.408	−2.552∼1.736	0.708	–	–	–
Pneumonia	2.805	−0.892∼6.501	0.137	–	–	–
Intraoperative tracheobronchial manifestations						
One manifestation	4.727	2.013∼7.442	0.001	4.741	2.194∼7.289	<0.001
Two manifestations	9.327	5.954∼12.700	<0.001	7.687	4.142∼11.231	<0.001
Three manifestations	10.336	2.787∼17.885	0.007	8.053	0.480∼15.627	0.037

Univariate analysis is on the left, and multivariate analysis is on the right.

## 4 Discussion

This study reveals that the majority (84.8%) of the patients fall within the age range of 1–3 years, with a higher prevalence in males (63.7%) compared to females (36.3%), consistent with previous research (1, 2). Children can aspirate various types of tracheobronchial foreign body, which can be broadly categorized into organic and inorganic matter. In our study, organic foreign body accounted for 94.8% of cases, predominantly consisting of items such as peanuts, walnuts, and melon seeds—aligning with prior findings (6–8). The preference for the location of tracheobronchial foreign body in children remains a subject of debate. Some studies suggest a higher frequency in the right bronchi (1, 7, 9), while others argue in favor of the left bronchi (10, 11), and some report a roughly equal distribution between left and right (2). In our study, 91.7% of children presented with unilateral foreign body, with no significant disparity in proportions between the left and right sides. This might be attributed to the theory proposed by Cleveland et al. (12), which suggests that most children under the age of 15 have similar angles and diameters in both their left and right bronchi, leading to nearly equivalent incidences of foreign body aspiration on either side.

Given these demographic characteristics, understanding the types and distribution of foreign body is crucial for clinical management. Regarding specific types of organic foreign body, peanuts were the most commonly encountered (36.5% of cases), followed by walnuts (26.1% of cases). Despite the theoretical differences in their physical and chemical properties, we did not find significant differences in surgical duration or complication rates among these different types of organic foreign body. This finding provides practical guidance for clinical management, suggesting that the type of organic foreign body may not be a crucial factor in predicting procedural difficulty. However, when comparing organic with inorganic foreign body, we observed that organic materials generally showed a greater tendency to cause mucosal inflammation. This difference likely stems from two key characteristics of organic materials: their hygroscopic nature allowing them to absorb moisture and expand in the airways, and their potential to release inflammatory mediators through degradation. These properties may trigger more pronounced local tissue reactions through both mechanical and chemical pathways. For instance, expanded organic materials can exert increased pressure on the airway mucosa, while their degradation products may stimulate local inflammatory responses. This observation aligns with the basic pathophysiological principle that organic materials typically induce more complex tissue responses compared to inert inorganic foreign body.

Our analysis revealed that the duration of foreign body retention was not found to be significantly correlated with surgical complications or procedural duration. This finding differs from traditional assumptions that longer retention times necessarily lead to more severe complications. This unexpected observation might be explained by several factors. First, we observed considerable variation in individual inflammatory responses among children, with some showing rapid inflammatory changes while others demonstrated minimal tissue reaction despite longer retention periods. Second, the specific position and degree of obstruction caused by the foreign body appeared to influence local tissue reactions more than the duration of retention itself. For instance, some partially obstructing foreign bodies were associated with relatively mild local changes despite longer retention times, while tightly wedged foreign bodies sometimes caused more severe tissue reactions within shorter periods. Third, the inflammatory response in some cases appeared to stabilize after the initial period, suggesting that the severity of local tissue reaction does not necessarily progress linearly with time. These observations suggest that comprehensive clinical assessment and bronchoscopic findings, rather than retention duration alone, may be more reliable indicators for surgical planning.

In this study, surgical durations ranged from 3 to 60 min, with a median time of 10.00 min (7.00, 15.00). This operative duration compares favorably with previous reports. For instance, Wang et al. (7) documented surgical durations ranging from 5 to 85 min with a median of 20 min in their retrospective analysis. Similarly, Li et al. (13) reported a mean operative time of 25.6 min in their cases. The relatively shorter median duration in our series might be attributed to our center's standardized surgical protocols, and experienced surgical teams. Our surgical protocol emphasizes efficient pre-operative planning, standardized instrument preparation, and coordinated teamwork between surgeons and anesthesiologists, which collectively contribute to optimizing procedural efficiency. However, we observed that certain cases required extended operative times, particularly those with complications such as significant granulation tissue or purulent exudates. Similar findings have been reported by Chen et al. (3), who noted that cases with inflammatory changes often required additional procedural time, because inflammatory changes and granulation tissue formation significantly increased procedural complexity. In our study, cases involving multiple tracheobronchial manifestations (such as mucosal hyperemia, edema, and granulation tissue) showed significantly longer operative times. These findings align with Gao et al.'s (8) observations about the impact of multiple airway manifestations on operative complexity. In cases with mucosal hyperemia and edema, reduced bronchoscopic visibility necessitates more careful and time-consuming manipulation. When purulent exudates are present, thorough suctioning and airway clearing are required before foreign body removal can be attempted. The cumulative effect of multiple manifestations compounds these challenges, explaining the progressive increase in operative times. While these extended operative times are sometimes necessary for safe foreign body removal in complicated cases, it is important to note that prolonged surgical procedures may increase the risk of postoperative adverse events. Studies by Li et al. (13) and Tan et al. (14) have demonstrated significant associations between extended surgical duration and increased postoperative adverse events and hospital stays.

Given the critical impact of surgical duration on postoperative adverse events, the choice of anesthesia management strategy plays a pivotal role in optimizing surgical conditions while maintaining patient safety. Two primary anesthesia management approaches are currently employed in pediatric tracheobronchial foreign body removal: maintaining spontaneous breathing vs. administering muscle relaxants (13). Maintaining spontaneous breathing preserves the child's respiratory drive and protective reflexes such as coughing, potentially reducing the risk of respiratory depression during the procedure. However, this approach may result in less stable surgical conditions and increased risk of airway injury due to patient movement. Conversely, the use of muscle relaxants provides superior surgical exposure and operating conditions, potentially shortening surgical duration, but requires more stringent respiratory management and carries the risk of residual muscle relaxation leading to postoperative respiratory depression. Based on our previous experience, anesthesiologists select the appropriate approach based on individual patient factors including age, comorbidities, and foreign body location. For younger children or those with respiratory infections or increased airway reactivity, maintaining spontaneous breathing is often preferred. In older children with good general condition, muscle relaxants may facilitate smoother surgical progression. While our current study did not include detailed analysis of these specific approaches, future prospective studies comparing these techniques would provide valuable insights into their impact on procedural efficiency and perioperative outcomes.

In this study, our multiple linear regression analysis identifies ASA III classification, pulmonary rales, and tracheobronchial manifestations—specifically mucosal hyperemia and edema, purulent exudate, and granulation tissue—as significant risk factors for prolonging the surgical duration of tracheobronchial foreign body removal in children.

The ASA physical status classification is widely used as an anesthesia risk assessment tool and is valuable for predicting perioperative complications and adverse events (15). A large multi-institutional study using the ACS NSQIP-P database found that higher ASA class (III or IV) was significantly associated with postoperative adverse events and prolonged hospital stay after pediatric bronchoscopy for foreign body removal (14). In our study, 3.3% children fell into the ASA III category due to poor preoperative physical conditions, unique foreign body characteristics, and an elevated estimated risk associated with anesthesia and surgery. Our study highlights that children with an ASA III classification experience an approximately 7.3-min increase in surgical duration compared to their ASA II counterparts. This prolonged duration is an expected finding, as ASA III patients typically require additional anesthetic procedures and interventions to ensure patient safety. These necessary anesthetic procedures may include: more frequent airway assessment and management, careful titration of anesthetic depth, additional airway suctioning and longer intervals between surgical steps for vital sign stabilization. For example, in one ASA III case, the patient suddenly experienced breath-holding, cyanosis, and respiratory distress during the procedure. Intraoperative monitoring revealed a significant drop in blood oxygen saturation, coupled with absent breath sounds upon auscultation. As anticipated for an ASA III patient, this situation required immediate additional interventions - we had to temporarily suspend the surgical procedure, perform thorough airway assessment, and implement closed thoracic drainage to address the pneumothorax. Similarly, in another ASA III case, the patient demonstrated heightened airway reactivity preoperatively. During surgery, instrument stimulation, such as from the bronchoscope, led to sustained increases in airway pressure and marked hypoxemia. This anticipated complication in an ASA III patient with known heightened airway reactivity necessitated specific anesthetic management procedures, including immediate suspension of surgical manipulation, deepening of anesthesia, administration of bronchodilators, and careful titration of positive pressure ventilation until oxygen saturation normalized. These examples emphasize that children with higher ASA physical status classifications face significant challenges during anesthesia and surgery, requiring predictable additional anesthetic interventions that inevitably contribute to prolonged surgical procedures. Therefore, meticulous preoperative preparation, comprehensive anesthesia planning, and careful intraoperative management are essential, particularly for cases with ASA III and above.

Pulmonary rales are important clinical indicators in the assessment of pediatric patients. Wet rales typically signify the passage of gas through tracheobronchi filled with inflammatory exudates, while dry rales result from gas turbulence through narrowed lumens. Our study indicates that some children present with preoperative clinical manifestations, including both dry and wet pulmonary rales. Notably, children with preoperative pulmonary rales experienced surgical durations approximately 6.8 min longer than those without rales. This finding highlights the significance of preoperative pulmonary rales as an independent risk factor for prolonged surgical duration in rigid bronchoscopic foreign body removal. Previous studies have consistently demonstrated that the presence of pulmonary rales complicates the removal of foreign body (6, 16). This complication may stem from the gradual expansion, rupture, and deterioration of plant-based foreign body in a humid and warm airway environment, along with heightened airway stimulation due to free fatty acids. These processes lead to mucosal hyperemia and edema, increased exudate, and further narrowing of the ventilation passage within an already obstructed lumen. Consequently, bronchoscopic visibility diminishes, and instrument manipulation becomes more challenging.

Additionally, our study identified various tracheobronchial manifestations through bronchoscopy, including mucosal hyperemia and edema, purulent exudate, and granulation tissue. These findings complicate the diagnosis and treatment of foreign body cases. Significant purulent exudates and pronounced granulation tissue can completely encase the foreign body, necessitating extended surgical durations to remove pus and clear the granulation. In instances of severe granulation tissue proliferation where the foreign body cannot be easily extracted, additional interventions—such as laser, electric scalpel, or snare—may be required to address local airway issues before foreign body removal. The process of removing granulation tissue itself can lead to localized bleeding, which poses the risk of airway obstruction if substantial. Moreover, airway edema, granulation, and scarring may contribute to bronchial contracture strictures, further complicating surgical procedures (17–19). Our findings demonstrate that children exhibiting one, two, or three tracheobronchial manifestations experienced increased surgical durations of approximately 4.7, 7.7, and 8.0 min, respectively, compared to those without any tracheobronchial manifestations. This underscores the notion that foreign body retention triggers a cascade of inflammatory responses. Continuous stimulation of the tracheobronchial mucosa by inflammation results in evident tracheobronchial changes, significantly increasing the difficulty and duration of foreign body removal.

Despite the insights gained from this study, several limitations should be acknowledged. First, this research is a single-center retrospective study that only includes children undergoing rigid bronchoscopy. It does not encompass cases treated with fiberoptic bronchoscopy at our institution, which may introduce bias into the results. Future large-scale studies involving multiple centers across different regions would provide more robust evidence. Second, although anesthetic management strategies (spontaneous breathing vs. muscle relaxants) may influence procedural outcomes, our initial study protocol primarily focused on preoperative parameters, without incorporating detailed anesthetic variables. As our understanding evolved during the study, the potential significance of different anesthetic management strategies became increasingly apparent. A prospective study specifically designed to compare these approaches would better elucidate their impact on surgical efficiency and perioperative outcomes. Lastly, our study did not capture several important postoperative outcomes, such as hospitalization rates, 30-day readmission rates, and comprehensive long-term follow-up data, particularly for those with exceptionally prolonged surgical durations. These clinical outcomes could have provided valuable insights into the relationship between surgical duration and postoperative recovery. Future prospective studies should incorporate these important outcome measures along with systematic follow-up procedures to better assess the impact of prolonged surgical duration on both immediate and long-term prognosis.

## 5 Conclusions

Boys aged 1–3 years are at a high risk for tracheobronchial foreign body aspiration, with organic materials being the predominant type. Factors such as ASA classification III, the presence of pulmonary rales, and tracheobronchial manifestations observed during bronchoscopy are associated with prolonged surgical durations for foreign body removal. Extended surgical times increase the risk of perioperative complications that can hinder postoperative recovery. Therefore, thorough preoperative assessments and effective preparations for potential intraoperative emergencies are essential, particularly for patients classified as ASA III or those presenting with pulmonary rales. This preparation should include ensuring the availability of both single-lumen and double-lumen endotracheal tubes, anesthesia machines, and suction devices for prompt clearance of airway secretions. Additionally, access to electrocautery for hemostasis is crucial for managing intra-tracheal bleeding, and bronchodilators like albuterol aerosols are necessary for bronchospasm management. Cardiovascular emergency medications should also be readily available. These measures are vital for reducing both intraoperative and postoperative complications, thereby enhancing patient recovery and overall prognosis.

## Data Availability

The raw data supporting the conclusions of this article will be made available by the authors, without undue reservation.
